# Rectal Location and Postcolonoscopy Colorectal Cancer Outcomes

**DOI:** 10.1001/jamanetworkopen.2025.13391

**Published:** 2025-06-02

**Authors:** Charles J. Kahi, Laura J. Myers, Patrick O. Monahan, Barry C. Barker, Timothy E. Stump, Thomas F. Imperiale

**Affiliations:** 1 Department of Medicine, Richard L. Roudebush VA Medical Center, Indianapolis, Indiana; 2 Health Services Research & Development, Richard L. Roudebush VA Medical Center, Indianapolis, Indiana; 3 Indiana University School of Medicine, Indianapolis; 4 Department of Biostatistics and Health Data Science, Indiana University School of Medicine, Indianapolis; 5 The Regenstrief Institute, Indianapolis, Indiana

## Abstract

This cohort study examines whether there are survival differences among patients with postcolonoscopy colorectal cancer based on cancer location.

## Introduction

Postcolonoscopy colorectal cancers (PCCRCs; ie, cancers that are diagnosed after a colonoscopy that did not detect cancer) are a core measure of colonoscopy quality.^1,2^ Our group has previously reported a PCCRC prevalence of 6% in the Veterans Health Administration (VHA).^3^ PCCRCs have a higher predilection for the proximal colon, but recent evidence shows that the rectum is one of the most common locations when the colorectum is stratified into its individual segments.^4,5^ This is important because rectal cancer has specific features that differ from cancer elsewhere in the colon, including a higher propensity for locoregional invasion. To our knowledge, no studies have assessed whether rectal location is associated with cancer-specific mortality (CSM) and all-cause mortality (ACM) among patients with CRC detected 6 months to 3 years after colonoscopy (PCCRC-3y).

## Methods

The cohort study was approved as exempt research by the institutional review boards at Indiana University and by the Research and Development Committees of the Richard L. Roudebush VA Medical Center in Indianapolis, Indiana. The institutional review board approved a waiver of informed consent given the retrospective cohort design. The study followed the (STROBE) reporting guideline.

We conducted a secondary analysis of a VHA-Medicare administrative database that includes veterans with CRC diagnosed between January 1, 2003, and December 31, 2013. Patients whose colonoscopy occurred 6 months or less before CRC diagnosis were defined as detected CRC (DCRC), and those whose colonoscopy occurred 6 to 36 months prior to CRC diagnosis as PCCRC-3y.^3^ Individuals were censored at the time of death or December 31, 2018. We used Cox proportional hazards regression to model the time to ACM and CSM after CRC diagnosis. The analyses were stratified by location (rectum or right colon [cecum, ascending colon, transverse colon]) and whether the patient was classified as PCCRC-3y or DCRC.

R version 4.2.1 (R Foundation for Statistical Computing) was used for all analyses. Final analyses were performed in February 2025. Covariates with a 2-sided *P* ≤ .15 were included in multivariable models, and *P* < .05 was considered statistically significant.

## Results

Among 5248 rectal cancers, 289 (5.5%) were classified as PCCRC-3y and 4959 (94.4%) as DCRC. Demographic and clinical features are summarized in Table 1. Based on multivariable Cox proportional hazards analysis (Table 2), there was no significant difference in ACM and CSM between rectal PCCRC-3y and rectal DCRC, with adjusted hazard ratios (aHRs) of 1.13 (95% CI, 0.98-1.30; *P* = .09) and 1.09 (95% CI, 0.90-1.33; *P* = .38), respectively. There were also no significant differences in ACM and CSM between rectal PCCRC-3y and right colon PCCRC-3y (Table 2). There was a statistically significant, but numerically modest, higher ACM and CSM for rectal DCRC compared with right colon DCRC.

**Table 1.** Baseline Characteristics

Characteristic	Patients, No. (%)					*P* value
	Overall (N = 14 205)	Rectal PCCRC-3y (n = 289)	Rectal DCRC (n = 4959)	Right PCCRC-3y (n = 999)	Right DCRC (n = 7958)	
Age, y						
Mean (SD)	68.20 (9.01)	68.12 (8.41)	66.17 (8.88)	69.85 (8.22)	69.27 (8.98)	<.001
Median (IQR)	68.00 (61.00-76.00)	67.00 (61.00-75.00)	65.00 (60.00-73.00)	70.00 (64.00-77.00)	69.00 (62.00-77.00)	
Range	50.00-85.00	51.00-85.00	50.00-85.00	50.00-85.00	50.00-85.00	
Age group, y						
≥65	8582 (60.4)	172 (59.5)	2505 (50.5)	714 (71.5)	5191 (65.2)	<.001
<65	5623 (39.6)	117 (40.5)	2454 (49.5)	285 (28.5)	2767 (34.8)	
Sex						
Male	13935 (98.1)	286 (99.0)	4891 (98.6)	977 (97.8)	7781 (97.8)	.004
Female	270 (1.9)	3 (1.0)	68 (1.4)	22 (2.2)	177 (2.2)	
Race						
White	11508 (81.0)	239 (82.7)	4157 (83.8)	831 (83.2)	6281 (78.9)	<.001
Black	2413 (17.0)	43 (14.9)	703 (14.2)	150 (15.0)	1517 (19.1)	
Additional groups^a^	284 (2.0)	7 (2.4)	99 (2.0)	18 (1.8)	160 (2.0)	
Indicator for Hispanic ethnicity						
No	13385 (94.2)	269 (93.1)	4670 (94.2)	948 (94.9)	7498 (94.2)	.67
Yes	820 (5.8)	20 (6.9)	289 (5.8)	51 (5.1)	460 (5.8)	
Weighted Charlson score						
Mean (SD)	2.38 (2.64)	2.23 (2.31)	1.82 (2.30)	3.04 (2.94)	2.65 (2.74)	<.001
Median (IQR)	2.00 (0.00-3.00)	2.00 (1.00-3.00)	1.00 (0.00-3.00)	2.00 (1.00-4.00)	2.00 (1.00-4.00)	
Range	0.00-18.00	0.00-12.00	0.00-18.00	0.00-17.00	0.00-17.00	
Statin use						
No	8549 (60.2)	152 (52.6)	3347 (67.5)	437 (43.7)	4613 (58.0)	<.001
Yes	5655 (39.8)	137 (47.4)	1612 (32.5)	562 (56.3)	3344 (42.0)	
NSAID use						
No	12522 (88.2)	240 (83.0)	4373 (88.2)	854 (85.5)	7055 (88.7)	.001
Yes	1682 (11.8)	49 (17.0)	586 (11.8)	145 (14.5)	902 (11.3)	
Aspirin use						
No	10476 (73.7)	204 (70.6)	3937 (79.4)	622 (62.3)	5713 (71.8)	<.001
Yes	3728 (26.2)	85 (29.4)	1022 (20.6)	377 (37.7)	2244 (28.2)	
Family history of CRC						
No	13914 (98.0)	285 (98.6)	4861 (98.0)	975 (97.6)	7793 (97.9)	.71
Yes	290 (2.0)	4 (1.4)	98 (2.0)	24 (2.4)	164 (2.1)	
Current smoker						
No	8738 (61.5)	167 (57.8)	2759 (55.6)	652 (65.3)	5160 (64.8)	<.001
Yes	5466 (38.5)	122 (42.2)	2200 (44.4)	347 (34.7)	2797 (35.1)	
AJCC cancer stage						
I	4825 (34.0)	114 (39.4)	1854 (37.4)	358 (35.8)	2499 (31.4)	<.001
II	3990 (28.1)	70 (24.2)	1232 (24.8)	251 (25.1)	2437 (30.6)	
III	3242 (22.8)	67 (23.2)	1083 (21.8)	217 (21.7)	1875 (23.6)	
IV	2148 (15.1)	38 (13.1)	790 (15.9)	173 (17.3)	1147 (14.4)	
Chemotherapy						
No	8353 (58.8)	122 (42.2)	1911 (38.5)	707 (70.8)	5613 (70.5)	<.001
Yes	5852 (41.2)	167 (57.8)	3048 (61.5)	292 (29.2)	2345 (29.5)	
Radiation therapy						
No	11253 (79.2)	132 (45.7)	2224 (44.8)	991 (99.2)	7906 (99.3)	<.001
Yes	2952 (20.8)	157 (54.3)	2735 (55.2)	8 (0.8)	52 (0.7)	
Immunotherapy						
No	14116 (99.4)	286 (99.0)	4927 (99.4)	993 (99.4)	7910 (99.4)	.74
Yes	89 (0.6)	3 (1.0)	32 (0.6)	6 (0.6)	48 (0.6)	
Surgery						
No	2204 (15.5)	71 (24.6)	1374 (27.7)	93 (9.3)	666 (8.4)	<.001
Yes	12001 (84.5)	218 (75.4)	3585 (72.3)	906 (90.7)	7292 (91.6)	

Abbreviations: AJCC, American Joint Committee on Cancer; CRC, colorectal cancer; DCRC, colorectal cancer detected with colonoscopy; NSAID, nonsteroidal anti-inflammatory drug; PCCRC-3y, colorectal cancer diagnosed 6 months to 3 years after colonoscopy did not detect cancer.

^a^ Includes Asian individuals as well as those with other or unknown race.

**Table 2.** Multivariable Cox Proportional Hazards Model of Time to All-Cause and CRC-Specific Death^a^

Comparison	Univariable		Multivariable			
			Excluding stage variable from model		Including stage variable in model	
	HR (95% CI)	*P* value	HR (95% CI)	*P* value	HR (95% CI)	*P* value
**Rectum, PCCRC-3y vs DCRC**						
All cause	1.22 (1.06-1.41)	.005	1.10 (0.96-1.27)	.17	1.13 (0.98-1.30)	.09
CRC specific	1.10 (0.91 1.34)	.32	1.06 (0.87-1.29)	.54	1.09 (0.90-1.33)	.38
**PCCRC-3y, rectum vs right**						
All cause	1.15 (0.98-1.34)	.08	0.96 (0.77-1.20)	.72	1.13 (0.91-1.42)	.27
CRC specific	1.41 (1.13-1.76)	.003	0.97 (0.70-1.36)	.87	1.26 (0.90-1.76)	.17
**DCRC, rectum vs right**						
All cause	1.06 (1.02-1.11)	.006	1.01 (0.95-1.07) 1.02	.75	1.07 (1.01-1.14)	.03
CRC specific	1.40 (1.31-1.49)	<.001	0.99 (0.91-1.08)	.84	1.11 (1.01-1.21)	.03

Abbreviations: CRC, colorectal cancer; DCRC, colorectal cancer detected with colonoscopy; HR, hazard ratio; PCCRC, colorectal cancer diagnosed 6 months to 3 years after colonoscopy did not detect cancer.

^a^ All models include the following covariates: age, sex, race, ethnicity, comorbidity, nonsteroidal anti-inflammatory use, aspirin use, family CRC history, smoking status, chemotherapy treatment, radiation treatment, immunotherapy treatment, and surgery treatment. Two sets of models were run, excluding and including the cancer stage variable.

## Discussion

We found comparable ACM and CSM between rectal PCCRC-3y and DCRC and between rectal PCCRC-3y and right colon PCCRC-3y. Overall, rectal location and classification as PCCRC-3y or DCRC appeared to have minimal to no impact on mortality after CRC diagnosis. These findings align with those of most studies assessing PCCRC-3y outcomes, although recent work suggests that conventional survival analyses of PCCRC are susceptible to lead time and immortal time biases.^6^ Additional study with PCCRCs defined after index colonoscopy is warranted.

The rectum has long been identified as an area that is prone to missed colorectal neoplasms. These are often located on the proximal side of the valves of Houston and can escape detection without careful examination. The association of PCCRCs with proximal colonic location has contributed to the current recommendation to perform a second examination of the right colon. However, this should not be at the detriment of other areas of the colon, and the fact that the rectum is a common location for PCCRC underscores the importance of meticulous and repeated inspection of this area.

Limitations of our study include missing or limited data on certain covariates (body mass index, physical activity, colonoscopy quality), potentially incomplete capture of colonoscopies done outside the VHA, and residual confounding. Our findings should be validated in more contemporary samples, ideally coupled with root-cause analyses to determine the factors contributing to the burden of rectal PCCRC.

## Notes

Supplement.
Data Sharing Statement
