# Supplementary material for: Rectal Location and Postcolonoscopy Colorectal Cancer Outcomes
Source: JAMA Netw Open. 2025 Jun 2;8(6):e2513391. doi: 10.1001/jamanetworkopen.2025.13391 (PMC12131095; doi:10.1001/jamanetworkopen.2025.13391)
Supplement: Supplement. — Data Sharing Statement [file jamanetwopen-e2513391-s001.pdf]

Data Sharing Statement

Kahi. Rectal Location and Postcolonoscopy Colorectal Cancer Outcomes. *JAMA Netw Open*. Published June 02, 2025. doi:10.1001/jamanetworkopen.2025.13391

Data
Data available: No
